# Identification of Key Pathways and Genes in the Dynamic Progression of HCC Based on WGCNA

**DOI:** 10.3390/genes9020092

**Published:** 2018-02-14

**Authors:** Li Yin, Zhihui Cai, Baoan Zhu, Cunshuan Xu

**Affiliations:** 1 College of Life Science, Henan Normal University, Xinxiang 453007, Henan, China; 2 State Key Laboratory Cultivation Base for Cell Differentiation Regulation and Henan Engineering Laboratory for Bioengineering and Drug Development, Henan Normal University, Xinxiang 453007, Henan, China; 3 Luohe Medical College, Luohe 462002, Henan, China; yourgravity@163.com (Z.C.); vvtong@yeah.net (B.Z.)

**Keywords:** hepatocellular carcinoma, WGCNA, time serial expression analysis, cell cycle, oxidative metabolism, Kaplan-Meier Survival analysis

## Abstract

Hepatocellular carcinoma (HCC) is a devastating disease worldwide. Though many efforts have been made to elucidate the process of HCC, its molecular mechanisms of development remain elusive due to its complexity. To explore the stepwise carcinogenic process from pre-neoplastic lesions to the end stage of HCC, we employed weighted gene co-expression network analysis (WGCNA) which has been proved to be an effective method in many diseases to detect co-expressed modules and hub genes using eight pathological stages including normal, cirrhosis without HCC, cirrhosis, low-grade dysplastic, high-grade dysplastic, very early and early, advanced HCC and very advanced HCC. Among the eight consecutive pathological stages, five representative modules are selected to perform canonical pathway enrichment and upstream regulator analysis by using ingenuity pathway analysis (IPA) software. We found that cell cycle related biological processes were activated at four neoplastic stages, and the degree of activation of the cell cycle corresponded to the deterioration degree of HCC. The orange and yellow modules enriched in energy metabolism, especially oxidative metabolism, and the expression value of the genes decreased only at four neoplastic stages. The brown module, enriched in protein ubiquitination and ephrin receptor signaling pathways, correlated mainly with the very early stage of HCC. The darkred module, enriched in hepatic fibrosis/hepatic stellate cell activation, correlated with the cirrhotic stage only. The high degree hub genes were identified based on the protein-protein interaction (PPI) network and were verified by Kaplan-Meier survival analysis. The novel five high degree hub genes signature that was identified in our study may shed light on future prognostic and therapeutic approaches. Our study brings a new perspective to the understanding of the key pathways and genes in the dynamic changes of HCC progression. These findings shed light on further investigations.

## 1. Introduction

Hepatocellular carcinoma (HCC) is one of the most common cancers worldwide and the second leading cause of global cancer-related death accounting for around 11% of all cancer deaths [1]. Chronic viral hepatitis, metabolic disease, autoimmune hepatitis are major risk factors for HCC development. Hepatitis C viruses (HCV) can cause acute and chronic infections which can lead to liver cirrhosis and hepatocellular carcinoma. Cirrhosis is the most important risk factor for developing HCC, and the majority of viral-associated HCC cases develop from liver cirrhosis, so the clarity of hepatitis viral infections in HCC development is necessary for the treatment of HCC [2]. It is estimated that there are 140 million infections with hepatitis C worldwide and the most-affected regions are Central and East Asia and North Africa. HCV-associated carcinogenesis can lead to increased hepatocyte proliferation and steatosis, oxidative stress, mitochondrial damage and induction of reactive oxygen species (ROS) [3]. Patients who have progressed to liver cirrhosis and poor liver function commonly develop HCC [4].

At present, the potentially curative options for HCC patients are radiofrequency ablation, liver transplantation and tumor resection. However, many factors affect the suitability, including tumor stage, deficiency of donors, graft rejection and opportunistic infections due to immunosuppression, etc. [5]. The liver cancer stage is one of the most important factors in choosing treatment options and predicting a patient’s outlook. For early stage HCC without cirrhosis, liver resection or liver transplantation is available, but recurrence is frequently occurred. At the end stage of HCC, patients often have less than three months survival. It is necessary to understand the mechanism of progression from cirrhosis to HCC.

Weighted gene co-expression network analysis (WGCNA) has been proved to be an effective method to detect co-expressed modules and hub genes, microRNAs and lncRNAs (long non-coding RNAs) in many aspects [6,7,8,9,10,11,12,13,14,15]. WGCNA can group genes into a model or network based on pairwise correlations between genes due to their similar expression profile, and these models can correlate to different stages of HCC. First, the absolute value of the correlation of paired genes was used to define the gene co-expression network. Next, an adjacency matrix is used to define the strength with wich genes are connected to each other. A soft thresholding parameter was employed to construct a weighted network. WGCNA uses the topological overlap measure (TOM) as a proximity measure to cluster genes into network modules that combine the adjacency of two genes and the connection strengths with which these two genes interact with other neighbor genes. The genes inside a module can be summarized with the module eigengene, which is defined as the first principal component of the expression profiles. To find the modules related to clinical traits of interest, the correlation is calculated between module eigengenes and all clinical traits. The correlation between genes and module eigengenes was used to identify intramodular hub genes [16].

In order to explore the dynamic changes during the development of HCC, we analyzed 75 tissue samples representing the stepwise carcinogenic process from pre-neoplastic lesions to HCC including normal, cirrhosis without HCC, cirrhosis, low-grade dysplastic, high-grade dysplastic, very early HCC, early HCC, advanced HCC and very advanced HCC stages by using WGCNA (Table S1). To the best of our knowledge, this is the first report describing the dynamics of gene expression changes during the development of HCC, though there are some studies about each stage of HCC based on the dataset [17,18].

In this study, we constructed a gene co-expression network based on WGCNA and identified 25 modules during the progression of HCC. The modules were correlated with the progression of HCC. We also performed canonical pathway analysis by Ingenuity pathway analysis (IPA) on five modules closely related to different HCC stages. The turquoise module enriched in the cell cycle which was activated only at four neoplastic stages, and the degree of the activity of the cell cycle corresponded to the deterioration degree of HCC. The orange and yellow modules enriched in energy metabolism, especially oxidative metabolism, and decreased only at four neoplastic stages. The brown module enriched in protein ubiquitination and ephrin receptor-signaling pathways which decrease at very early stages of HCC only. The darkred module enriched in hepatic fibrosis/hepatic stellate cell activation, which isactivated at the cirrhotic stage only. Then, we performed upstream regulator analysis and detected some regulators such as PPARA (Peroxisome Proliferator Activated Receptor Alpha ) and PLG (Plasminogen). We also identified the high degree genes in every module using the cytohubba plugin based on cytoscape [19].

## 2. Materials and Methods

### 2.1. Data Processing

The gene expression dataset GSE6764 provided by Wurmbach, E. et al. [17] (https://www.ncbi.nlm.nih.gov/geo/query/acc.cgi?acc=GSE6764) was downloaded from the Gene Expression Omnibus (GEO) database [20]. In total, 75 tissue samples were divided into eight consecutive pathological stages from pre-neoplastic lesions to HCC including normal, cirrhosis without HCC, cirrhosis, low-grade dysplastic, high-grade dysplastic, very early HCC, early HCC, advanced HCC and very advanced HCC. All tissue samples are hybridized on the human U133 plus 2.0 array (Affymetrix, Santa Clara, CA, USA). The Robust Multi-array Average (RMA) algorithm was performed to process the raw files. In order to filter the features exhibiting little variation across the above samples, the *nsFilter* algorithm was used to filter the data for the subsequent WGCNA [16,21].

### 2.2. Construction of Weighted Gene Co-Expression Networks and Identification of Modules Associated with Different Stages of Hepatocellular Carcinoma

From thousands of genes, the interesting gene modules can be identified by WGCNA, and then, the intramodular connectivity and gene significance based on the correlation of a gene expression profile with a sample trait were used to identify key genes in HCC for further validation. WGCNA is a freely accessible R package for the construction of weighted gene co-expression networks [22]. The above filtered data were used to construct the network. Three different ways can be selected to construct the network and identify modules according to different needs. In our study, the one-step function was used for network construction and detection of consensus modules.

### 2.3. Interaction Analysis of Co-Expression Modules

To further evaluate the co-expression similarity of all the modules, the eigengenes adjacency based on their correlation was calculated. The interaction relationship among different co-expression modules was performed by the flashClust function [23]. A heat map was used for visualization of the correlations of each module.

### 2.4. Functional Enrichment Analysis of Genes in Every Module

Hub gene is a loosely defined term which is an abbreviation of “highly connected gene”. The genes inside co-expression modules have high connectivity and the genes within the same module may play similar roles. We filtered the hub genes in each module according to the intra-modular connectivity and correlation with module eigengenes. To identify the biological function of the significant modules and the relationship between the modules and different stages, we extracted the top ranked genes with the strongest connections within each module to perform canonical pathways analysis in selected modules using of IPA.

### 2.5. The Ingenuity Pathway Analysis Upstream Regulator Analysis

The co-expressed genes participating in the same biological process or disease may be regulated by the same or similar regulators especially transcription factors (TF). In order to explain the biological activities of each module, we identified the upstream transcriptional regulators in each module with a *p* value of overlap <0.01.

### 2.6. Protein-Protein Interaction Network Construction and Analysis for Selected Modules

The top ranked genes in every module are thought to be hub genes. In order to identify the high degree genes which play a critical role in the protein-protein network (PPI), the *Cytohubba* plugin based on Cytoscape was used to perform the network analysis [19], and the high degree genes were identified.

### 2.7. Kaplan-Meier Survival Analysis

Publicly available data and tools were employed to perform the survival analysis using the The Cancer Genome Atlas (TCGA)-liver cancer data which contained 361 samples with the high degree hub genes as input.

For the duplicated genes, all probe sets/records will be averaged per sample using quantile-normalized data. The maximum risk groups were selected for the survival analysis. All the details are described in the tutorial provided on the SurvExpress website [24].

## 3. Results

### 3.1. Data Processing

A total of 75 tissue sample raw files (.CEL format) were downloaded from the NCBI (National Center for Biotechnology Information). The raw files were converted to expression data using the RMA algorithm based on R language including background correction, normalization and summarization. There were a total of 16,383 probes for further WGCNA analysis after *nsFilter* processing.

### 3.2. Construction of Weighted Gene Co-Expression Network Identification of Modules Associated with Different Stages of HCC

**Figure 1:**
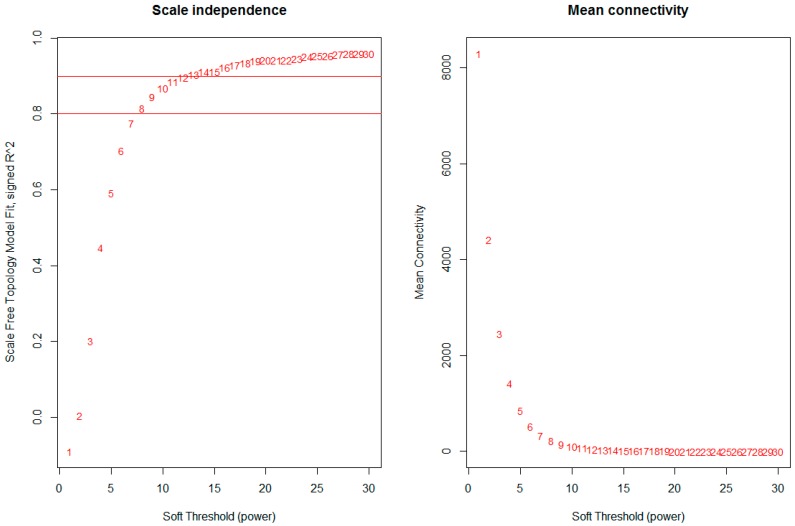
Network topology for different soft-thresholding powers. Numbers in the plots indicate the corresponding soft thresholding powers. The approximate scale-free topology can be attained at the soft-thresholding power of 8.

**Figure 2:**
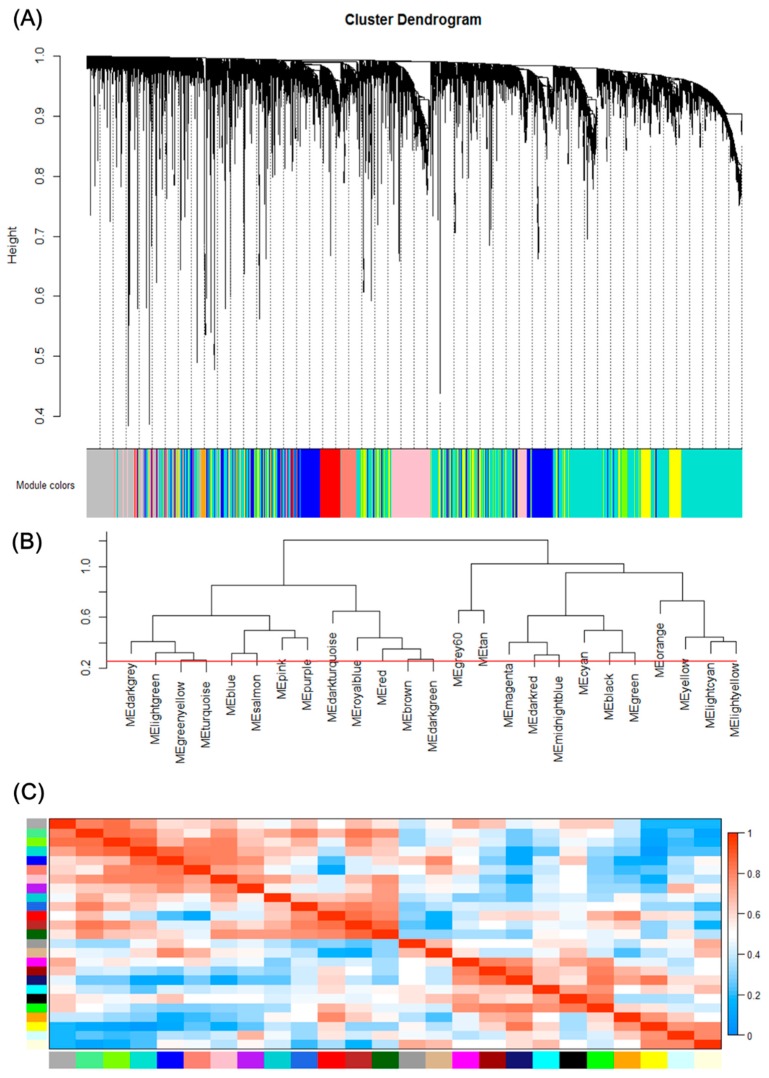
Gene modules identified by Weighted gene co-expression network analysis (WGCNA). (**A**) Gene dendrogram obtained by clustering the dissimilarity based on consensus Topological Overlap with the corresponding module colors indicated by the color row. Each colored row represents a color-coded module which contains a group of highly connected genes. A total of 25 modules were identified. (**B**) Dendrogram of consensus module eigengenes obtained by WGCNA on the consensus correlation. The red line is the merging threshold, and groups of eigengenes below the threshold represent modules whose expressions profiles should be merged due to their similarity. (**C**) Heatmap plot of the adjacencies of modules. Red represents high adjacency (positive correlation) and blue represents low adjacency (negative correlation).

The network was constructed from the filtered probes and twenty-five modules were identified. We have chosen the soft threshold power 8 to define the adjacency matrix based on the criterion of approximate scale-free topology (Figure 1), with minimum module size 30, the module detection sensitivity *deepSplit* 2, and cut height for merging of modules 0.2 which means that the modules whose eigengenes are correlated above 0.8 will be merged (Figure 2A).

### 3.3. Correlation between each Module

**Figure 3:**
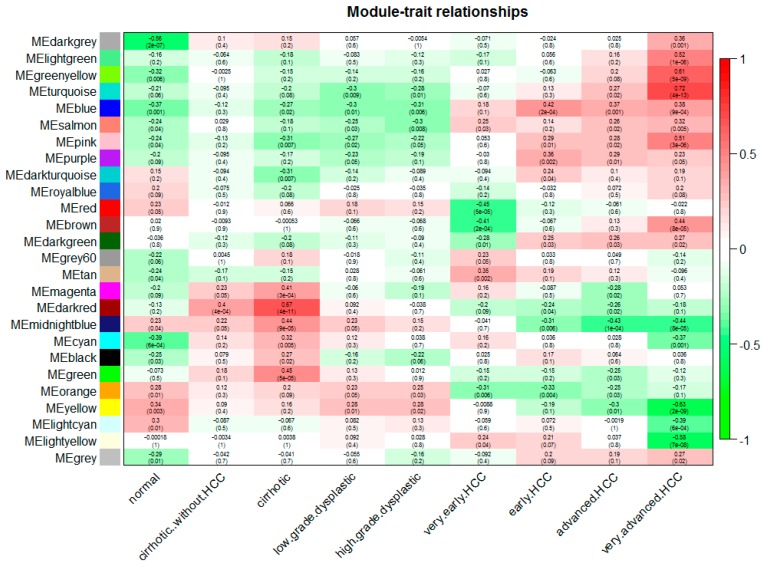
Relationships of consensus module eignegenes and different stages of hepatocellular carcinoma (HCC). Each row in the table corresponds to a consensus module, and each column to a stage. The module name is shown on the left side of each cell. Numbers in the table report the correlations of the corresponding module eigengenes and stage, with the *p* values printed below the correlations in parentheses. The table is color coded by correlation according to the color legend. Intensity and direction of correlations are indicated on the right side of the heatmap (red, positively correlated; green, negatively correlated).

We can find that some of the modules had similar expression profiles. In order to figure out the interactions among these 25 co-expressed modules, the connectivity of eigengenes was analyzed. As shown in Figure 2B,C, we performed a cluster analysis. In general, 25 clusters were grouped into two clusters, and each cluster contains three branches. Combined with Figure 3, there was a significant difference among the 25 modules. However, no pair of modules below the threshold (0.2) was merged. There are multiple modules related to one or more tumor stages. For instance, the turquoise, light green, green yellow, blue, salmon, pink and purple modules were related to four tumor stages, especially the turquoise module, which is strongly related to the development of HCC; the red and brown modules were negatively related to the very early stage; the tan modules were specifically related to the very early stage; the magenta, darkred, cyan, black and green modules were related to the pre-tumor stage; the midnight blue, orange, yellow and light cyan modules were negatively related to four HCC stages.

### 3.4. Functional Enrichment Analysis

**Figure 4:**
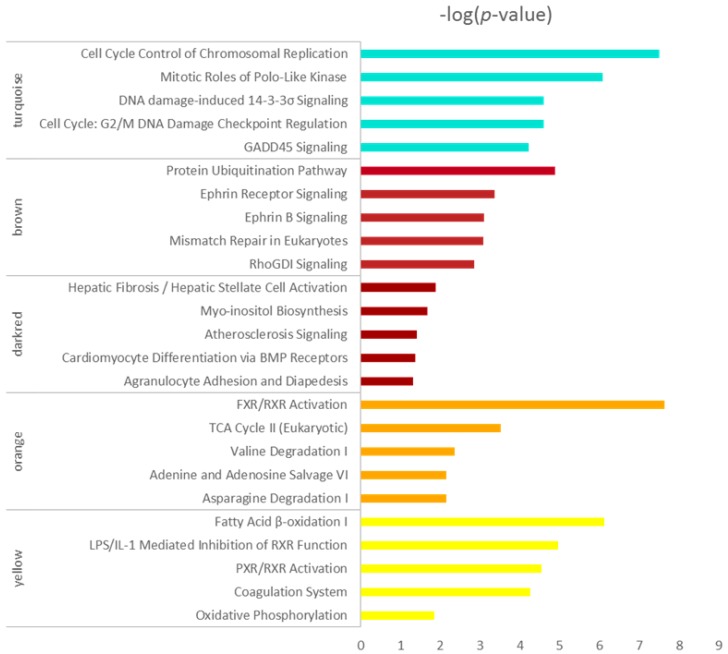
Top 5 enrichment results of canonical pathway analysis by ingenuity pathway analysis (IPA) for co-expressed genes in turquoise, brown, darkred, orange and yellow modules. Pathway names are shown on the left, and the bars on the right represent the −log(*p* value) of the corresponding pathway. The different colors of the bars are in accordance with the corresponding modules.

Five modules intimately related to different HCC stages were selected for the canonical pathway analysis by IPA, and the hub genes in the five modules are listed in the supplementary table. As shown in Figure 4, the enriched pathways including cell cycle, mitosis, DNA damage-induced 14-3-3σ signaling, G2/M DNA damage checkpoint regulating and GADD45 signaling, mainly in the turquoise module, indicated that the cell cycle-related pathways changed initially at very early HCC and changed more significantly as the disease worsened, which is in agreement with previous studies [25,26,27]. The biological activity enriched in the brown module reduced suddenly at the very early stage of HCC, which included the protein ubiquitination pathway, etc. The pathways in the darkred module were activated in the cirrhotic tissue samples and decreased in four stages of HCC which mainly include hepatic fibrosis/hepatic stellate cell activation. The orange and yellow modules were all inactivated at four stages of HCC, as shown in Figure 4; the enrichment pathways participated mainly in metabolism. The energy metabolism changed significantly. The process related to oxidative metabolism decreased in HCC progression which indicated the alternation of the supply method of energy.

### 3.5. The Ingenuity Pathway Analysis Upstream Regulator Analysis

Upstream regulator analysis was performed for the genes from selected modules using IPA. As shown in Table 1, several kinds of upstream regulators were predicted including transcription regulators, transporters, microRNA, growth factors and enzymes, etc. The ligand-dependent nuclear receptor PPARA was identified in turquoise and yellow modules at the same time with different target molecules. *PLG* and *GPD1* are both hub genes in the orange and yellow module respectively.

### 3.6. PPI Network Construction and Analysis of Selected Modules

**Figure 5:**
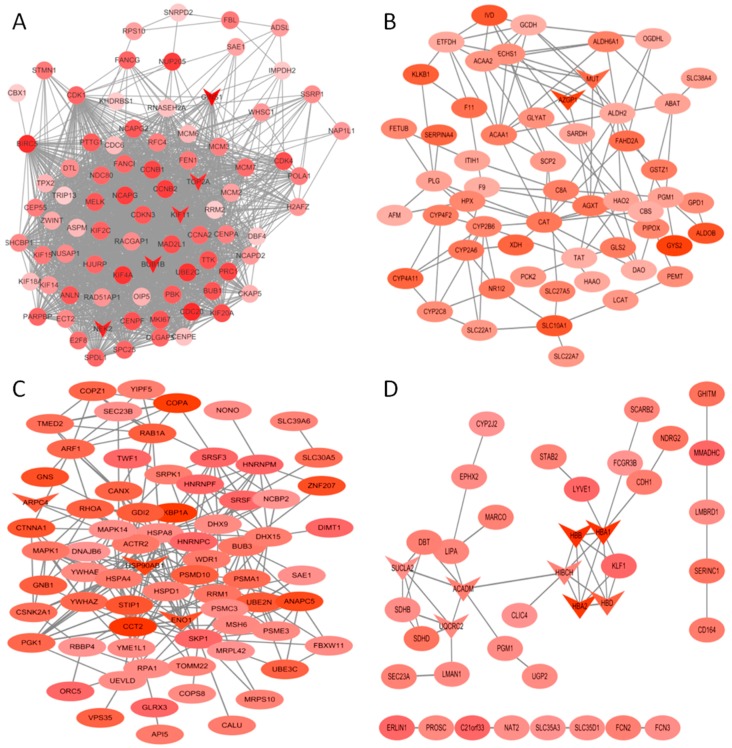
Protein-protein interaction (PPI) network of genes in the turquoise (**A**), yellow (**B**), brown (**C**) and orange (**D**) modules. The density of the ellipse corresponds to module membership (kME) values. The network was constructed using Cytoscape 3.4 software. The genes with a v-shape represent the high-degree genes from cytohubba.

The co-expression networks of top ranked genes for four selected modules, including the turquoise, brown, orange and yellow module were constructed as shown in Figure 5. The high degree genes calculated by the *cytohubba* plugin are shown in a “v” shape. *GINS1*, *NEK2*, *BUB1B*, *KIF11* and *TOP2A* were identified in the turquoise module, which was enriched in cell cycle-related processes. *MUT*, *AZGP1*, *HBB*, *HBA1*, *HBA2*, *HBD*, *SUCLA2*, *ACADM* and *UQCRC2* were identified in the yellow and orange modules, which were enriched in oxidative metabolism. *KBP1A*, *ARPC4*, *HSP90AB1* and *ENO1* were high degree genes involved in the early stage of HCC.

### 3.7. Kaplan-Meier Survival Analysis

Kaplan-Meier Survival analysis was performed to determine the relationship between the expression of high degree hub genes and the survival time of HCC patients.

**Figure 6:**
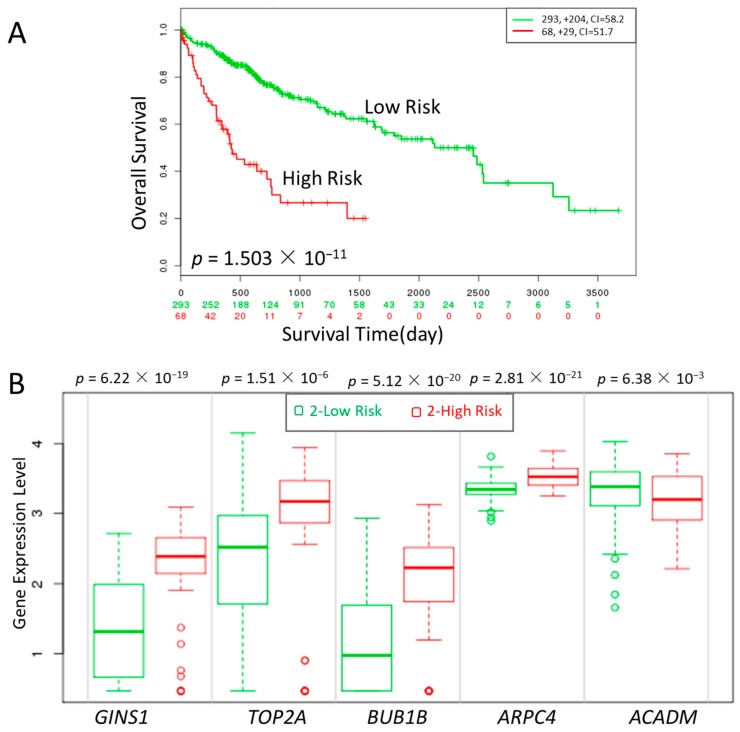
Kaplan-Meier curves of gene groups (*GINS1*, *TOP2A*, *BUB1B*, *ARPC4*, *ACADM*) in The Cancer Genome Atlas (TCGA) liver cancer dataset based on SurvExpress (*n* = 381). “+” marks on the upper figure represents censoring samples. Horizontal axis represents time (day) to event. Outcome event, time scale, concordance index (CI) and *p* value of the log-rank test are shown. Red and green curves represent High- and Low-risk groups, respectively. The number below the horizontal axis represents the number of individuals not presenting the event of the corresponding risk groups over time. (**A**) The xpression of five genes is correlated with high risk, poor prognosis and shorter overall survival time. (**B**) box plot of the five genes across risk groups with the *p* value.

Three cell-cycle related genes (*GINS*, *BUB1B* and *TOP2A*), one oxidative metabolism-related gene (*ACADM*) and one early stage-related gene (*ARPC4*) were significantly correlated with high risk, poor prognosis and shorter overall survival, respectively, as shown in Figure 6. The result showed that the identified genes were able to distinguish the high risk from low risk patients effectively.

## 4. Discussion

As a global health problem, HCV can cause infections which lead to liver cirrhosis and HCC. The multivariate analysis of risk factors for HCC has been extensively documented [2,3,28]. The main objectives of the study were to gain molecular insights into the progression of HCC and to identify and predict the candidate gene groups associated with the stepwise carcinogenic process from pre-neoplastic lesions to HCC by constructing a gene co-expression network using WGCNA. The significantly changed modules that correlated with different stages of HCC were identified, and the genes in the same module were extracted to perform pathway enrichment analysis. Then, the upstream regulators and hub genes were identified by using integrated bioinformatics methods including cytoscape and IPA.

Modules changed significantly at four neoplastic stages included the turquoise, orange and yellow modules. The cell cycle-associated turquoise module changed significantly in all stages of carcinogenesis, and the change was exacerbated as the disease worsened. All above results suggest that gene expression associated with cell cycle changed at the very early stage of HCC. It is well known that the cell cycle, the process of cell progression and division lies at the heart of cancer. Cells use a complex set of kinases to control the complex steps in the cell cycle. Once the regulatory process malfunctions, uncontrolled cell proliferation occurs. Checkpoints are important quality control measures, which ensure proper cell cycle events. Therefore, regulation of the cell cycle could be used to cure cancer especially the cell cycle checkpoint [29,30,31,32,33]. It can be seen that from Figure 3, the activation of the genes associated with the cell cycle occurred at the very early stage of HCC.

All this indicated that the abnormal cell cycle-associated pathways in the transcriptome play a critical role in the initiation and development of HCC. The orange and yellow modules, enriched in energy metabolism, especially oxidative metabolism, decreased only in four neoplastic stages. In addition, the biological process decreased significantly in the very advanced stage. All this suggested the switch of an altered energy source, which is consistent with recent studies [34,35,36,37,38,39,40]. Otto Warburg thought that the core metabolic signature of cancer cells is a high glycolytic flux, and the prime cause of cancer is the replacement of respiration of oxygen by glycolysis due to defective mitochondrial respiration [40]. As the energy and redox currencies of the cell, ATP and NADH are key factors for tumor survival, growth, and expansion, which makes them the core for therapeutic exploitation. Our findings are consistent with this opinion. We found a decrease in the tricarboxylic acid (TCA) cycle, fatty acid ß-oxidation and oxidative phosphorylation in all stages of HCC, which indicated a decrease in oxygen consumption. What is the primary origin of cancer; genetic mutations or energetic imbalances? We are not sure.

According to the PPI network analysis from the selected models, some high-degree hub genes were identified which played critical roles in the network. For the turquoise module, *GINS1*, *TOP2A*, *KIF11*, *BUB1B* and *NEK2* were identified asthe high degree genes. For yellow and orange modules, *MUT*, *AZGP1*, *HBA1*, *HBB*, *HBD*, *HBA2*, *ACADM*, *UQCRC2* and *SUCLA2* were high degree genes.

*GINS1* (also known as *PSF1*) is a subunit of the GINS (Go, lchi, Nii, San) complex which drives the unwinding of DNA in front of the replication fork [41]. Many studies have shown that *GINS1* is up-regulated in several cancer types including lung, colon, prostate, colorectal and breast cancers [41,42,43,44,45,46,47]. It is reported that *GINS1* is expressed widely in early embryogenesis in mice, stem and progenitor cells, etc. [42,48,49]. All the above showed a close relationship between *GINS1* and the cell cycle. From previous results, high *GINS1* expression correlated with a more aggressive phenotype as well as worse prognosis in HCC patients [50]. Through the analysis of HCC tissue, *TOP2A* expressions were correlated with advanced histological grading, microvascular invasion and an early age onset of the malignancy [51,52] which indicated the prognostic value of *TOP2A* in HCC. As for *KIF11* and BUB1B, there are relatively few reports on their function in HCC, but all of them are related to the cell cycle [53,54,55,56,57,58,59,60,61]. Amazingly, *HBA1*, *HBB*, *HBD* and *HBA2* all appeared in the yellow and orange modules, which are related to energy metabolism. It is well known that the tumor cells have a high glycolytic rate compared to normal cells even if oxygen is sufficient [62]. It is assumed that impaired mitochondrial respiration may play a vital role in the process [63].There are some reports on the relationship between hemoglobin and HCC, but the mechanism is not well understood [64,65]. Are there some correlations between the deregulation of hemoglobin and altered energy metabolism? This is worth further study. Increasing evidence has shown that the decrease of zinc-alpha2-glycoprotein (*AZGP1*) is associated with poor prognosis and more aggressive tumors in HCC [66,67]. And according to a recent study, *AZGP1* plays a role in regulating the PTEN/Akt and CD44 pathways [61]. As important enzymes in the electron transport chain (ETC), the roles of *UQCRC2* and *SUCLA2* are not known yet.

The brown module was enriched in protein ubiquitination and ephrin receptor signaling pathways which decrease at the very early stage of HCC only. Eph receptors belong to receptor tyrosine kinase families and participate in many cancers. A previous study has shown that EphrinA2 suppressed apoptosis, rather than accelerated proliferation and facilitated cancer cell survival [68]. In the present study, ephrin signaling deregulated at the very early stage, and upregulated increasingly with the progression of HCC. According to the network analysis of the brown module, *ARPC4*, *HSP90AB1*, *ENO1* were identified as high degree hub genes. As a subunit of the actin-related protein 2/3 complex (ARP2/3), ARPC4 may contribute to the development of HCC [69]. It can be inferred that some biological processes occurred at all stages of HCC and some at a certain stage, which shows the complexity of the development of HCC. The darkred module, enriched in hepatic fibrosis/hepatic stellate cell activation, that was correlated with the cirrhotic stage only, will be further studied in the future.

The survival analysis showed that the five novel high-degree hub gene signatures identified in our selected modules which changed significantly may shed light on future prognostic and therapeutic approaches.

In conclusion, we have presented a novel approach using WGCNA to explore the dynamic changes during the stepwise carcinogenic process from pre-neoplastic lesions to HCC including eight stages. According to the network constructed by WGCNA, 25 modules were identified, and five modules were selected to be analyzed in detail. We found that the turquoise module, enriched in the cell cycle-related process, was activated at all stages of HCC, and the yellow and orange module, enriched in aerobic metabolism, was inactivated in four stages of HCC. Some hub genes with a high degree were identified. All of the above may shed light on the understanding of pathways and genes underlying HCV-associated disease and the application of prognostic and predictive markers for HCC, which needed further analysis.

## Figures and Tables

**Table 1:** Upstream regulators of selected modules predicted by IPA.

Module	Upstream Regulator	Type	*p* Value of Overlap	Target Molecules in Dataset
turquoise	*MYC*	transcription regulator	3.94 × 10^−10^	*CCNB1, CCNB2, CDC20, CDK1, MCM6*
	*MED1*	transcription regulator	1.06 × 10^−9^	*BIRC5, CCNB1, CDC20, CDK1, CDK4, CENPA*
	*FOXM1*	transcription regulator	0.000308	*CCNB2, CDC20*
	*ATP7B*	transporter	0.000373	*CDC20, PTTG1, TRIP13*
	*CNR1*	g-protein coupled receptor	0.00157	*CCNB2, CDK1*
	*PPARA*	ligand-dependent nuclear receptor	0.00521	*CCNB1, CDK1, CDK4, H2AFZ, TOP2A*
	*TOB1*	transcription regulator	0.00538	*HJURP, SPDL1*
brown	*NFE2L2*	transcription regulator	0.00662	*ARF1, HSP90AB1, PSMA1, PSMC3, STIP1, TMED2*
	*LMF1*	other	0.00911	*CANX*
	*mir-451*	microRNA	0.00911	*YWHAZ*
orange	*LEP*	growth factor	0.00714	*ACADM, EPHX2, LIPA, UGP2*
	*PLG*	peptidase	0.00787	*CLIC4, STAB2*
yellow	*PPARA*	ligand-dependent nuclear receptor	7.66 × 10^−8^	*ACAA1, ALDH2, ALDOB, C8A, CAT, CYP2B6, CYP2C8, CYP4A11, GPD1, GSTZ1, HPX, SCP2*
	*GPD1*	enzyme	0.00000244	*C8A, CYP2B6, CYP2C8, CYP39A1, CYP4A22, F11*
	*SLC25A13*	transporter	0.00000244	*C8A, CYP2B6, CYP2C8, CYP39A1, CYP4A22, F11*
	*FECH*	enzyme	0.0000416	*CYP2B6, CYP2C8, CYP4A11, SLC10A1*
	*RORC*	ligand-dependent nuclear receptor	0.0000458	*CYP2A6 (includes others), CYP2B6, CYP2C8, CYP39A1, CYP4A11, RDH16*
	*RORA*	ligand-dependent nuclear receptor	0.0000485	*CYP2A6 (includes others), CYP2B6, CYP2C8, CYP39A1, CYP4A11, RDH16*
	*LEP*	growth factor	0.0000887	*ALDOB, CYP2C8, CYP4A11, PEMT, SCP2, SLC27A5*
	*EHHADH*	enzyme	0.000106	*ACAA1, CYP4A11, SCP2*
	*HSD17B4*	enzyme	0.000106	*ACAA1, CYP4A11, SCP2*
	*ACOX1*	enzyme	0.000492	*ACAA1, CAT, CYP4A11*
	*POR*	enzyme	0.000531	*CYP2A6 (includes others), CYP2B6, CYP2C8, CYP39A1, CYP4A11*
	*TCF7L2*	transcription regulator	0.00056	*ACAA1, CYP2C8, GYS2*
	*AHR*	ligand-dependent nuclear receptor	0.000667	*ALDH2, CYP2B6, CYP2C8, RDH16, SLC22A7*
	*NR1H4*	ligand-dependent nuclear receptor	0.000878	*CYP2B6, LCAT, NR1I2, SLC10A1*
	*HNF4A*	transcription regulator	0.0011	*CAT, HPX, NR1I2, SCP2, SLC10A1*
	*ZBTB20*	transcription regulator	0.00172	*CYP2B6, CYP2C8, GHR*
	*NR1I3*	ligand-dependent nuclear receptor	0.00186	*CYP2A6 (includes others), CYP2B6, CYP2C8, CYP39A1*
	*STAT1*	transcription regulator	0.00344	*CYP2C8, CYP4A11*
	*STAT6*	transcription regulator	0.004	*CIDEB, CYP4A11*
	*ABCC4*	transporter	0.00687	*CYP2B6*
	*IL25*	cytokine	0.00687	*CIDEB*
	*TERC*	other	0.00894	*CYP2C8, CYP4A11*

## Supplementary Materials

The following are available online at http://www.mdpi.com/2073-4425/9/2/92/s1.

## References

[1] Ferlay J., Soerjomataram I., Dikshit R., Eser S., Mathers C., Rebelo M., Parkin D.M., Forman D., Bray F. (2015). Cancer incidence and mortality worldwide: Sources, methods and major patterns in globocan 2012. Int. J. Cancer.

[2] Ikeda K., Saitoh S., Koida I., Arase Y., Tsubota A., Chayama K., Kumada H., Kawanishi M. (1993). A multivariate analysis of risk factors for hepatocellular carcinogenesis: A prospective observation of 795 patients with viral and alcoholic cirrhosis. Hepatology.

[3] Ringehan M., McKeating J.A., Protzer U. (2017). Viral hepatitis and liver cancer. Philos. Trans. R. Soc. Lond. B. Biol. Sci..

[4] Dhungel B., Jayachandran A., Layton C.J., Steel J.C. (2017). Seek and destroy: Targeted adeno-associated viruses for gene delivery to hepatocellular carcinoma. Drug Deliv..

[5] Waghray A., Murali A.R., Menon K.N. (2015). Hepatocellular carcinoma: From diagnosis to treatment. World J. Hepatol..

[6] Bo L., Wei B., Wang Z., Kong D., Gao Z., Miao Z. (2017). Screening of critical genes and microRNAs in blood samples of patients with ruptured intracranial aneurysms by bioinformatic analysis of gene expression data. Med. Sci. Monit..

[7] Guo X., Xiao H., Guo S., Dong L., Chen J. (2017). Identification of breast cancer mechanism based on weighted gene coexpression network analysis. Cancer Gene Ther..

[8] Huang H., Zhang Q., Ye C., Lv J.M., Liu X., Chen L., Wu H., Yin L., Cui X.G., Xu D. (2017). Identification of prognostic markers of high grade prostate cancer through an integrated bioinformatics approach. J. Cancer Res. Clin. Oncol..

[9] Liu X., Hu A.X., Zhao J.L., Chen F.L. (2017). Identification of key gene modules in human osteosarcoma by co-expression analysis weighted gene co-expression network analysis (WGCNA). J. Cell Biochem..

[10] Ma C., Lv Q., Teng S., Yu Y., Niu K., Yi C. (2017). Identifying key genes in rheumatoid arthritis by weighted gene co-expression network analysis. Int. J. Rheum. Dis..

[11] Tang Y., Ke Z.P., Peng Y.G., Cai P.T. (2018). Coexpression analysis reveals key gene modules and pathway of human coronary heart disease. J. Cell. Biochem..

[12] Esposti D.D., Hernandez-Vargas H., Voegele C., Fernandez-Jimenez N., Forey N., Bancel B., Le Calvez-Kelm F., McKay J., Merle P., Herceg Z. (2016). Identification of novel long non-coding RNAs deregulated in hepatocellular carcinoma using RNA-sequencing. Oncotarget.

[13] Giulietti M., Occhipinti G., Principato G., Piva F. (2017). Identification of candidate miRNA biomarkers for pancreatic ductal adenocarcinoma by weighted gene co-expression network analysis. Cell Oncol..

[14] Liao X., Huang K., Huang R., Liu X., Han C., Yu L., Yu T., Yang C., Wang X., Peng T. (2017). Genome-scale analysis to identify prognostic markers in patients with early-stage pancreatic ductal adenocarcinoma after pancreaticoduodenectomy. Oncol. Targets Ther..

[15] Giulietti M., Occhipinti G., Principato G., Piva F. (2016). Weighted gene co-expression network analysis reveals key genes involved in pancreatic ductal adenocarcinoma development. Cell Oncol..

[16] Langfelder P., Horvath S. (2008). WGCNA: An R package for weighted correlation network analysis. BMC Bioinformatics.

[17] Wurmbach E., Chen Y.B., Khitrov G., Zhang W., Roayaie S., Schwartz M., Fiel I., Thung S., Mazzaferro V., Bruix J. (2007). Genome-wide molecular profiles of HCV-induced dysplasia and hepatocellular carcinoma. Hepatology.

[18] Zheng S., Tansey W.P., Hiebert S.W., Zhao Z. (2011). Integrative network analysis identifies key genes and pathways in the progression of hepatitis C virus induced hepatocellular carcinoma. BMC Med. Genom..

[19] Chin C.H., Chen S.H., Wu H.H., Ho C.W., Ko M.T., Lin C.Y. (2014). cytoHubba: Identifying hub objects and sub-networks from complex interactome. BMC Syst. Biol..

[20] Barrett T., Wilhite S.E., Ledoux P., Evangelista C., Kim I.F., Tomashevsky M., Marshall K.A., Phillippy K.H., Sherman P.M., Holko M. (2013). Ncbi Geo: Archive for Functional Genomics Data Sets--Update. Nucleic Ac. Res..

[21] Irizarry R.A., Hobbs B., Collin F., Beazer-Barclay Y.D., Antonellis K.J., Scherf U., Speed T.P. (2003). Exploration, normalization, and summaries of high density oligonucleotide array probe level data. Biostatistics.

[22] R Core Team (2014). R: A Language and Environment for Statistical Computing. R Foundation for Statistical Computing.

[23] Langfelder P., Horvath S. (2012). Fast R Functions for Robust Correlations and Hierarchical Clustering. J. Stat. Softw..

[24] Aguirre-Gamboa R., Gomez-Rueda H., Martinez-Ledesma E., Martinez-Torteya A., Chacolla-Huaringa R., Rodriguez-Barrientos A., Tamez-Pena J.G., Trevino V. (2013). SurvExpress: An online biomarker validation tool and database for cancer gene expression data using survival analysis. PLoS ONE.

[25] Aarts M., Linardopoulos S., Turner N.C. (2013). Tumour selective targeting of cell cycle kinases for cancer treatment. Curr. Opin. Pharmacol..

[26] Diaz-Moralli S., Tarrado-Castellarnau M., Miranda A., Cascante M. (2013). Targeting cell cycle regulation in cancer therapy. Pharmacol. Ther..

[27] Williams G.H., Stoeber K. (2012). The cell cycle and cancer. J. Pathol..

[28] Liccioni A., Reig M., Bruix J. (2014). Treatment of hepatocellular carcinoma. Dig. Dis..

[29] Benada J., Macurek L. (2015). Targeting the checkpoint to kill cancer cells. Biomolecules.

[30] Broustas C.G., Lieberman H.B. (2014). DNA damage response genes and the development of cancer metastasis. Radiat. Res..

[31] Marques S., Fonseca J., Silva P.M., Bousbaa H. (2015). Targeting the spindle assembly checkpoint for breast cancer treatment. Curr. Cancer Drug Targets.

[32] Wang D.H., Guo L., Wu X.H. (2015). Checkpoint inhibitors in immunotherapy of ovarian cancer. Tumour Biol..

[33] Wang H., Zhang X., Teng L., Legerski R.J. (2015). DNA damage checkpoint recovery and cancer development. Exp. Cell. Res..

[34] Amann T., Hellerbrand C. (2009). GLUT1 as a therapeutic target in hepatocellular carcinoma. Expert Opin. Ther. Targets.

[35] Amann T., Maegdefrau U., Hartmann A., Agaimy A., Marienhagen J., Weiss T.S., Stoeltzing O., Warnecke C., Scholmerich J., Oefner P.J. (2009). GLUT1 expression is increased in hepatocellular carcinoma and promotes tumorigenesis. Am. J. Pathol..

[36] Beyoglu D., Idle J.R. (2013). The metabolomic window into hepatobiliary disease. J. Hepatol..

[37] Dong T., Yan Y., Chai H., Chen S., Xiong X., Sun D., Yu Y., Deng L., Cheng F. (2015). Pyruvate kinase M2 affects liver cancer cell behavior through up-regulation of HIF-1alpha and BCL-xL in culture. Biomed. Pharmacother..

[38] Kimhofer T., Fye H., Taylor-Robinson S., Thursz M., Holmes E. (2015). Proteomic and metabonomic biomarkers for hepatocellular carcinoma: A comprehensive review. Br. J. Cancer.

[39] Liu A.M., Xu Z., Shek F.H., Wong K.F., Lee N.P., Poon R.T., Chen J., Luk J.M. (2014). miR-122 targets pyruvate kinase M2 and affects metabolism of hepatocellular carcinoma. PLoS ONE.

[40] Oronsky B.T., Oronsky N., Fanger G.R., Parker C.W., Caroen S.Z., Lybeck M., Scicinski J.J. (2014). Follow the ATP: Tumor energy production: A perspective. Anticancer Agents Med. Chem..

[41] Kanzaki R., Naito H., Kise K., Takara K., Eino D., Minami M., Shintani Y., Funaki S., Kawamura T., Kimura T., Okumura M., Takakura N. (2016). PSF1 (partner of SLD five 1) is a prognostic biomarker in patients with non-small cell lung cancer treated with surgery following preoperative chemotherapy or chemoradiotherapy. Ann. Surg. Oncol..

[42] Nagahama Y., Ueno M., Miyamoto S., Morii E., Minami T., Mochizuki N., Saya H., Takakura N. (2010). PSF1, a DNA replication factor expressed widely in stem and progenitor cells, drives tumorigenic and metastatic properties. Cancer Res..

[43] Wen J.Z., Han X.Y., Wei B., Zhang S., Wei H.B. (2013). Expression of PSF1 in colon cancer tissues and its effect on the proliferation of colon cancer cells. Zhonghua Wei Chang Wai Ke Za Zhi.

[44] Zhang J., Wu Q., Wang Z., Zhang Y., Zhang G., Fu J., Liu C. (2015). Knockdown of PSF1 expression inhibits cell proliferation in lung cancer cells in vitro. Tumour Biol..

[45] Nakahara I., Miyamoto M., Shibata T., Akashi-Tanaka S., Kinoshita T., Mogushi K., Oda K., Ueno M., Takakura N., Mizushima H. (2010). Up-regulation of PSF1 promotes the growth of breast cancer cells. Genes Cells.

[46] Tahara H., Naito H., Kise K., Wakabayashi T., Kamoi K., Okihara K., Yanagisawa A., Nakai Y., Nonomura N., Morii E. (2015). Evaluation of PSF1 as a prognostic biomarker for prostate cancer. Prostate Cancer Prostatic Dis..

[47] Wei H.B., Wen J.Z., Wei B., Han X.Y., Zhang S. (2011). Expression and clinical significance of GINS complex in colorectal cancer. Zhonghua Wei Chang Wai Ke Za Zhi.

[48] Han Y., Ueno M., Nagahama Y., Takakura N. (2009). Identification and characterization of stem cell-specific transcription of PSF1 in spermatogenesis. Biochem. Biophys. Res. Commun..

[49] Ueno M., Itoh M., Kong L., Sugihara K., Asano M., Takakura N. (2005). PSF1 is essential for early embryogenesis in mice. Mol. Cell. Biol..

[50] Zhou L., Sun X.J., Liu C., Wu Q.F., Tai M.H., Wei J.C., Lei L., Meng F.D., Qu K., Xu J. (2015). Overexpression of PSF1 is correlated with poor prognosis in hepatocellular carcinoma patients. Int. J. Biol. Markers.

[51] Panvichian R., Tantiwetrueangdet A., Angkathunyakul N., Leelaudomlipi S. (2015). TOP2A amplification and overexpression in hepatocellular carcinoma tissues. Biomed. Res. Int..

[52] Wong N., Yeo W., Wong W.L., Wong N.L., Chan K.Y., Mo F.K., Koh J., Chan S.L., Chan A.T., Lai P.B. (2009). TOP2A overexpression in hepatocellular carcinoma correlates with early age onset, shorter patients survival and chemoresistance. Int. J. Cancer.

[53] Huang J., Wang L., Jiang M., Lin H., Qi L., Diao H. (2012). PTHLH coupling upstream negative regulation of fatty acid biosynthesis and Wnt receptor signal to downstream peptidase activity-induced apoptosis network in human hepatocellular carcinoma by systems-theoretical analysis. J. Recept. Signal Transduct. Res..

[54] Jin B., Wang W., Du G., Huang G.Z., Han L.T., Tang Y.Z., Fan D.G., Li J., Zhang S.Z. (2015). Identifying hub genes and dysregulated pathways in hepatocellular carcinoma. Eur. Rev. Med. Pharmacol. Sci..

[55] Qi L., Wang L., Huang J., Jiang M., Diao H., Zhou H., Li X., Jiang Z. (2013). Activated amelogenin Y-linked (AMELY) regulation and angiogenesis in human hepatocellular carcinoma by biocomputation. Oncol. Lett..

[56] Wang L., Huang J., Jiang M., Lin H., Qi L., Diao H. (2012). Activated PTHLH coupling feedback phosphoinositide to G-protein receptor signal-induced cell adhesion network in human hepatocellular carcinoma by systems-theoretic analysis. Sci. World J..

[57] Li G., Zhong Y., Shen Q., Zhou Y., Deng X., Li C., Chen J., Zhou Y., He M. (2017). NEk2 serves as a prognostic biomarker for hepatocellular carcinoma. Int. J. Oncol..

[58] Lin S., Zhou S., Jiang S., Liu X., Wang Y., Zheng X., Zhou H., Li X., Cai X. (2016). NEK2 regulates stem-like properties and predicts poor prognosis in hepatocellular carcinoma. Oncol. Rep..

[59] Wen S., Liu Y., Yang M., Yang K., Huang J., Feng D. (2016). Increased NEK2 in hepatocellular carcinoma promotes cancer progression and drug resistance by promoting PP1/Akt and Wnt activation. Oncol. Rep..

[60] Wubetu G.Y., Morine Y., Teraoku H., Yoshikawa M., Ishikawa D., Yamada S., Ikemoto T., Saito Y.U., Imura S., Shimada M. (2016). High NEK2 expression is a predictor of tumor recurrence in hepatocellular carcinoma patients after hepatectomy. Anticancer Res..

[61] Tian H., Ge C., Zhao F., Zhu M., Zhang L., Huo Q., Li H., Chen T., Xie H., Cui Y. (2017). Downregulation of AZGP1 by Ikaros and histone deacetylase promotes tumor progression through the PTEN/Akt and CD44s pathways in hepatocellular carcinoma. Carcinogenesis.

[62] Koppenol W.H., Bounds P.L., Dang C.V. (2011). Otto Warburg's contributions to current concepts of cancer metabolism. Nat. Rev. Cancer.

[63] Moreno-Sanchez R., Rodriguez-Enriquez S., Marin-Hernandez A., Saavedra E. (2007). Energy metabolism in tumor cells. FEBS J..

[64] Chen Y.L., Lin H.C., Lin K.H., Lin L.S., Hsieh C.E., Ko C.J., Hung Y.J., Lin P.Y. (2015). Low hemoglobin level is associated with the development of delirium after hepatectomy for hepatocellular carcinoma patients. PLoS ONE.

[65] Donadon V., Balbi M., Valent F., Avogaro A. (2010). Glycated hemoglobin and antidiabetic strategies as risk factors for hepatocellular carcinoma. World J. Gastroenterol..

[66] Huang Y., Li L.Z., Zhang C.Z., Yi C., Liu L.L., Zhou X., Xie G.B., Cai M.Y., Li Y., Yun J.P. (2012). Decreased expression of zinc-alpha2-glycoprotein in hepatocellular carcinoma associates with poor prognosis. J. Transl. Med..

[67] Xu M.Y., Chen R., Yu J.X., Liu T., Qu Y., Lu L.G. (2016). AZGP1 suppresses epithelial-to-mesenchymal transition and hepatic carcinogenesis by blocking TGFbeta1-ERK2 pathways. Cancer Lett..

[68] Feng Y.X., Zhao J.S., Li J.J., Wang T., Cheng S.Q., Yuan Y., Wang F., Wang X.F., Xie D. (2010). Liver cancer: EphrinA2 promotes tumorigenicity through Rac1/Akt/NF-kappaB signaling pathway. Hepatology.

[69] Qin G., Dang M., Gao H., Wang H., Luo F., Chen R. (2017). Deciphering the protein-protein interaction network regulating hepatocellular carcinoma metastasis. Biochim. Biophys. Acta.

